# Compact FTICR Mass Spectrometry for Real Time Monitoring of Volatile Organic Compounds

**DOI:** 10.3390/s18051415

**Published:** 2018-05-03

**Authors:** Joël Lemaire, Sébastien Thomas, Allan Lopes, Essyllt Louarn, Hélène Mestdagh, Hubert Latappy, Julien Leprovost, Michel Heninger

**Affiliations:** 1 Laboratoire de Chimie Physique, CNRS, Univ. Paris Sud, Université Paris Saclay, 91405 Orsay, France; allan.lopes@u-psud.fr (A.L.); essyllt.louarn@u-psud.fr (E.L.); helene.mestdagh@u-psud.fr (H.M.); Hubert_Latappy@leco.com (H.L.); 2 AlyXan, Centre Hoche, 91260 Juvisy-sur-Orge, France; sebastien.thomas@csnsm.in2p3.fr (S.T.); julien.leprovost@alyxan.fr (J.L.)

**Keywords:** chemical ionization, mass spectrometry, real time analysis, Fourier transform ion cyclotron resonance, direct quantification, volatile organic compounds

## Abstract

In this paper, we present a compact Fourier transform ion cyclotron resonance mass spectrometer (FTICR-MS) designed for real time analysis of volatile organic compounds (VOCs) in air or in water. The spectrometer is based on a structured permanent magnet made with NdFeB segments. Chemical ionization is implemented inside the ICR cell. The most widely used reaction is the proton transfer reaction using H_3_O^+^ precursor ions, but other ionic precursors can be used to extend the range of species that can be detected. Complex mixtures are studied by switching automatically from one precursor to another. The accuracy obtained on the mass to charge ratio (Δ*m*/*z* 5 × 10^−3^), allows a precise identification of the VOCs present and the limit of detection is 200 ppb without accumulation. The time resolution is a few seconds, mainly limited by the time necessary to come back to background pressure after the gas pulses. The real time measurement will be illustrated by the monitoring of VOCs produced during the thermal degradation of a polymer and by an example where three different precursor ions are used alternatively to monitor a gas sample.

## 1. Introduction

Fourier transform ion cyclotron resonance mass spectrometers (FTICR-MS) are generally known to be extremely heavy and expensive instruments implementing very high magnetic fields (up to 21 T), and needing a very low pressure in the chamber housing the ICR cell (10^−10^ mbar) obtained using many turbomolecular pumps [1]. Their fields of predilection involve the analysis of complex mixtures of high mass molecules such as in proteomics, in petroleomics [2], or for studies of dissolved organic matter [3].

The instruments we develop and present in this paper are in a very different field. They are small (compared to the previous), robust, easy-to-use field instruments for real-time detection of volatile organic compounds in air or water. As the molecules to be detected are in a lower mass range, it is possible to use weaker magnetic fields, of the order of 1 T [4,5,6,7,8,9]. This is sufficient to perform mass to charge measurement with an accuracy of Δ*m*/*z* 5 × 10^−3^, which allows differentiation of quasi-isobaric species having the same nominal mass but different molecular formulas. For instance, the signals associated to CO^+^ (*m*/*z* 27.995), N_2_^+^ (*m*/*z* 28.006), and C_2_H_4_^+^ (*m*/*z* 28.031) can be separated and therefore identified in a mixture.

Our instruments benefit from the fact that FTICR mass spectrometers are ion traps in which ions can be formed, manipulated, selected, reacted, and detected with a high mass resolution. During the detection, the vacuum must be good enough so that the motion of the ions is not too much affected by collisions. For this, the pressure at the time of detection must be of the order of 10^−8^ mbar. This requires that all gases used during the formation of ions or during the reaction with the sample are pulsed and have been pumped away at detection time. The design of the vacuum chambers and the pumping speed of the turbomolecular pump result in pump down times of the order of 200 ms. Thus, it makes possible measurements with a repetition rate close to one Hertz, which is well adapted for real-time monitoring. In this way, every few seconds, all the organic compounds present are detected, with no a priori knowledge on the species present and with a resolving power adapted to the monitoring of complex mixtures.

The ionization method implemented is crucial for trace detection and must allow a simple interpretation of the results given by mass spectrometry. The method of choice for real time analysis of complex and/or much diluted mixtures is chemical ionization (CI), a soft and selective ionization technique. CI is based on the use of a chemical reaction with a well-chosen ion to ionize the neutral compounds of interest present in the sample [10]. The resulting ionic products must be characteristic of the neutral analyte. Implementation of CI in the ICR cell and examples of CI reactions are described in Section 2.2. The ion-molecule reaction is performed under extremely well-defined reaction conditions (pressure, duration). The resulting mass spectra are then easy to analyze. The exact mass allows us to attribute a molecular formula to each detected species and the intensity of the peak is related to the mixing ratio of the compound in the sample. The only element required to obtain the concentration is the rate coefficient of the CI reaction. This rate is often known from experimental measurements, but if it is not the case, it can also be calculated with a good precision using known physical properties of the molecule [11].

Electron ionization (EI) is also possible and provides information on the main constituents of the matrix.

Prototype permanent magnet FTICR-MS are used in the LCP laboratory, either for kinetic studies of ion molecule reactions [12,13,14], for spectroscopic studies of ions in the infrared or the UV spectral regions [15,16], or for trace detection. A more compact version (Lxwxh = 65 × 72 × 104 cm^3^) is commercialized by the AlyXan company and is mainly applied to real time measurements.

## 2. Compact FTICR Mass Spectrometers

### 2.1. FTICR Detection

The detection of ions in an FTICR mass spectrometer consists of the following steps:

- Broadband excitation of the ion cyclotron motion
- Detection and digitalization of the image current induced by the ions
- Fourier transformation to obtain the frequency spectrum
- Change of the coordinate from frequency to mass using a calibration law

The broadband excitation signal must achieve excitation of all the ions of interest with equal efficiency. It can be either a chirp (a sinusoidal wave that increases in frequency linearly with time) or a SWIFT (stored waveform inverse Fourier transform, the signal is defined in the frequency domain and the corresponding time domain signal is obtained using inverse Fourier transform) [17]. The excitation is produced using an arbitrary waveform generator, followed by an amplifier and then a transformer producing two out of phase outputs.

One of the specificities of our instruments is the use of the same two electrodes to apply the excitation signal and to detect the ions. An optoelectronic low capacitance switch is used to connect them either to the excitation circuit or to the detection preamplifier [18].

At the end of the excitation, the ions of a given mass rotate in phase in the cell, on a large radius orbit. This coherent motion induces an image current in the detection circuit. If a single *m*/*z* is present, the image current is a sinusoid whose frequency is the ion cyclotron frequency and the amplitude is proportional to the number of ions.

If ions with different *m*/*z* are in the cell, the image current is a superposition of sinusoids and the Fourier transform gives the amplitude as a function of the cyclotron frequency for each ion packet. After the application of a calibration law to convert the frequency to mass, we obtain the mass spectrum.

**Figure 1:**
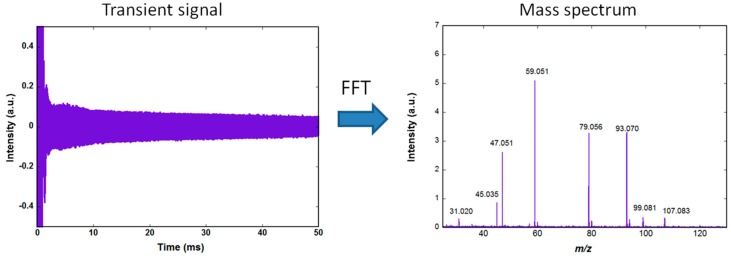
The ions are coherently excited by a broadband excitation appearing here as a saturation during the first ms of the signal. After the excitation, the ions rotate on large radius orbits in the cell and the image current induced in the detection circuit is amplified and recorded using a fast digitizer. A window function is applied to the transient (here a Hanning window) and a fast Fourier transform gives the frequency spectrum. The mass spectrum is obtained after rescaling of the abscissa using a calibration law.

Figure 1 shows the amplified image current, recorded using an Acquitek 350 PCI card (termed transient signal) and the corresponding mass spectrum obtained after Fourier transformation and conversion of the frequencies to mass. The acquisition is performed at a rate of 10 MS/s (Mega samples per second) using a 12 bits analog to digital converter. The same PCI card is used to generate the excitation signal. The slow decrease of the transient during the 50 ms observation time is due to dephasing of the ions motion resulting from collisions with background neutral molecules.

**Figure 2:**
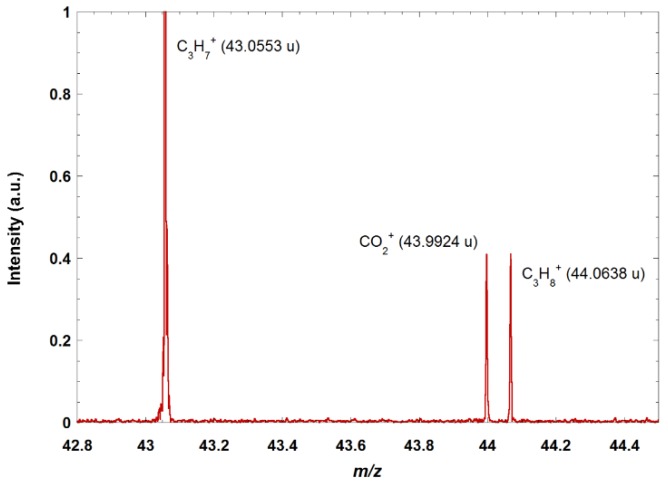
Zoom on *m*/*z* 43 and 44 of the mass spectrum obtained after electron ionization of a mixture of alkanes and CO_2_. The doublet at *m*/*z* 44 is attributed to C_3_H_8_^+^ (fragment ions coming from the electron ionization on propane and butane) and CO_2_^+^. The accuracy of the experimental mass to charge ratio measurement is Δ*m*/*z* 5 × 10^−3^. The resolving power Δm/m (with Δm calculated from the full width at half maximum of the peaks) is 10,000 for a 40 ms transient and no accumulation.

Figure 2 shows a zoom on a portion of the mass spectrum obtained when doing electron ionization on a mixture of several alkanes (methane, propane, butane) and CO_2_. The quasi-isobars at mass 44 are clearly resolved and identified.

### 2.2. Permanent Magnets

In FTICR mass spectrometers, the quality of the magnetic field is essential since the determination of the masses is based on the measurement of the cyclotron frequency of the ions. When only the magnetic field is present, the cyclotron frequency is given by:

(1) $$\omega =\frac{qB}{m}$$

where ω is the cyclotron frequency (in Hz), *q* is the charge (in C), *m* is the mass of the ion (in kg), and *B* is the intensity of the magnetic field (in T). Since *q* = ze (number of charge times the elementary charge), the cyclotron frequency is inversely proportional to *m*/*z*.

As the magnetic field is not the only field present in the ICR cell, a two term calibration law, of the form proposed by Ledford [19],

(2) $$\frac{m}{z}=\frac{a}{\omega }+\frac{b}{\omega^{2}}$$

is used, in which the first term corresponds to the effect of the magnetic field and the second term takes into account both the effect of the electrostatic trapping field in the cell and the influence of the space charge.

The magnetic field must be intense and homogeneous. With a higher nominal field, a larger number of ions can be trapped and the dynamics of the instrument are better (the maximum number of trapped ions is proportional to the square of the magnetic field) [20]. The cyclotron frequency of the ions is higher and the resolution power is better for fixed detection conditions.

In the FTICR spectrometers using superconducting magnets, fields in the range from 7 to 21 T are used and the relative homogeneity of the magnetic field in the cell is generally better than 1 ppm, measured in the central 1 cm diameter spherical volume. In the case of systems based on permanent magnets, the fields and the relative homogeneities are weaker. But there are many advantages in return: the system can be transported, there is no need for cryogenic fluids, the only requirement is an electrical power of 1 kW, and the spectrometer can be operated as soon as the vacuum is low enough. The cost is also much lower. The FTICR mass spectrometers using structured permanent magnets are robust field instruments. They do not target the same mass ranges or the same resolutions as the superconducting instruments.

**Figure 3:**
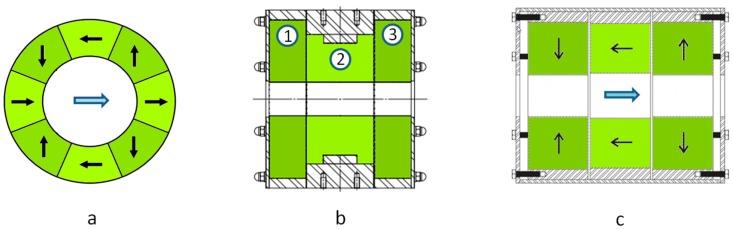
Different ways of generating an intense and homogeneous magnetic field using a permanent magnet assembly: (**a**) Cylindrical structure proposed by Klaus Halbach, here divided into eight segments. (**b**) Similar structure including three Halbach cylinders (**c**) Structure producing a field coaxial with the bore. The black arrows in (**a**,**c**) show the magnetization direction of the segments, while the blue arrows show the direction of the magnetic field produced in the bore.

Magnets based on a Halbach structure [21] are a very effective way to obtain a high field in the center of the bore of the magnet cylinder while having a low stray field outside. The sectional view of a Halbach structure composed of eight segments producing a dipolar field is shown in Figure 3a. When using off the shelf Halbach cylinders, made of Neodyme Iron Boron (NdFeB) with a bore diameter of 50 mm, it is possible to obtain a nominal field of ~1 T with a relative homogeneity of the order of 1% to 2%. It is possible to improve the nominal field and the homogeneity by using a more complex configuration in which several Halbach cylinders are associated. With the one shown in Figure 3b, incorporating three Halbach cylinders, we have obtained a nominal field as high as 1.5 T and a relative homogeneity improved to less than 0.5% for a magnet weighing 60 kg.

Another possible structure in which the field produced is coaxial with the magnet main axis is shown in Figure 3c. It is composed of three cylinders, two of which (the lateral ones) are magnetized radially, outwardly for one and inwardly for the other. The central cylinder helps in closing the magnetic field with a magnetization parallel to the axis. This structure is not as efficient as the previous one at producing an intense field. The maximum field will rather be of the order of 1 T for a 50 mm bore diameter and a weight of 50 kg. The relative homogeneity is 0.5%. The advantage of this configuration is that it is possible to move the ions along the cylinder axis. Thus, it is possible to form the ions outside the magnetic field and then guide them inside the ICR cell. We have used such a configuration to form the ions in a source operated at atmospheric pressure and then to transfer them into the vacuum chamber, trap them in an hexapole RF trap, and finally transfer them to the ICR cell to mass detect them.

### 2.3. Chemical Ionization

In our instruments, the ionization technique used is mainly chemical ionization. It is also possible to directly ionize a sample by electron ionization but this ionization method has two drawbacks with the analysis of VOCs at low concentration: (i) most of the ions are produced from the matrix (air or water) and the ions of interest are in very minor amounts; and (ii) electron ionization leads to spectra difficult to interpret because subsequent fragmentations occur.

Chemical ionization is based on ion-molecule reactivity [20]. The reactions are conducted at near thermal energy as the precursor ions can be relaxed by collisions. CI is a selective method, as only the targeted species are ionized thanks to the specific reaction, and it is considered as a soft ionization technique, as little to no fragmentation occurs.

#### 2.3.1. With Hydronium Ions: H_3_O^+^

The choice of the reacting ion, called the precursor, is decisive as it determines what type of molecules will be detected. In environmental analysis, the most used precursor is H_3_O^+^, which leads to the protonation of VOCs by the proton transfer reaction (PTR) [22,23,24]:

H_3_O^+^ + M → MH^+^ + H_2_O (3)

Hydronium ions do not react with the major components of air (N_2_, O_2_, Ar, CO_2_, CH_4_) as they have a proton affinity (PA) lower than water (691 kJ.mol^−1^ [25]), which means that reaction 1 is endothermic. This avoids saturating the ion trap and the detection of compounds present at trace level becomes possible. On the contrary, most VOCs have a PA larger than water, they are ionized by proton transfer, and the reaction occurs efficiently at almost every collision between H_3_O^+^ and a VOC molecule. Exceptions are alkanes and freons (CFC, HCFC, etc.) which have low PAs. Reactivity of VOCs with H_3_O^+^ generally leads to the exclusive formation of the protonated species. Fragmentation may be observed, for instance, the loss of a H_2_O molecule from protonated alcohols [26]. The mass spectrum is then easy to interpret as usually one peak corresponds to one compound present in the mixture. Moreover, exact masses obtained from FTICR measurements allow for identification of the molecular formulas [27].

In the ICR cell, chemical ionization is carried out under well-controlled conditions. Precursor ions are first prepared, and, since it is possible to combine different reactions as well as mass selection steps after electron ionization, a very large variety of ions can be prepared. Hydronium ions are prepared from water vapor in the cell after an electron ionization step:

H_2_O + e^−^(70 eV) → H_2_O^+•^ + 2 e^−^ (4)

The electron ionization produces H_2_O^+^ and also fragments ions OH^+^, O^+^, and H_2_^+^. These ions will further react on water vapor water to form H_3_O^+^.

OH^+^ + H_2_O → H_2_O^+•^ + OH^•^ (5)

O^+•^ + H_2_O → H_2_O^+•^ + O (6)

H_2_^+•^ + H_2_O → H_2_O^+•^ + H_2_ (7)

H_2_O^+•^ + H_2_O → H_3_O^+^ + OH^•^ (8)

**Figure 4:**
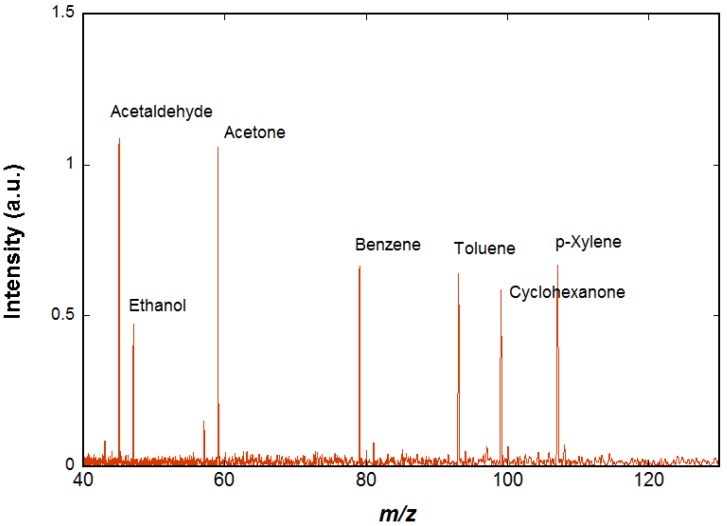
Reaction of H_3_O^+^ ions with a mixture of species diluted in air (acetaldehyde, ethanol, acetone, benzene, cyclohexane, toluene, cyclohexanone, p-xylene, and n-decane) is presented. The H_3_O^+^ ions do not react significantly with cyclohexane and n-pentane but proton transfer occurs with all the other species, as is observed on the mass spectrum.

The spectrum shown in Figure 4 is obtained using chemical ionization by H_3_O^+^ on a mixture of species (acetaldehyde, ethanol, acetone, benzene, cyclohexane, toluene, cyclohexanone, p-xylene, and n-decane) diluted in air at trace level (a few ppm). The H_3_O^+^ ions are prepared as described above. In order to ensure that H_3_O^+^ ions are the only ions present in the cell, a selection event is performed using an excitation signal containing all possible cyclotron frequencies except that of the H_3_O^+^ ions, so that all unwanted ions are ejected. A delay of 2 s is observed in order to let the H_3_O^+^ ions interact with the neutral molecules of the gas pulse and then to pump away the neutral molecules while all the ions, both precursors and reaction products, remain trapped in the cell by the magnetic and electric fields. These ions are detected when the pressure has returned to background level. Two of the species present in air, cyclohexane, and n-decane do not react significantly with H_3_O^+^ and therefore are not visible on the resulting mass spectrum. All the other species appear on the spectrum and the ions observed correspond to the protonated VOCs.

#### 2.3.2. Other Possible Precursors

The ICR cell gives us great flexibility for the preparation of precursor ions. The use of alternative precursors is useful for detecting species that are not ionized with H_3_O^+^ precursors or to increase selectivity. Small alkanes detection, for instance, can be conducted using CF_3_^+^. The precursor is formed from CF_4_ electron ionization. During the process, other minor ions are formed: CF_4_^+^, CF_2_^+^, and CF^+^. A mass selection is then carried out so as to keep only the CF_3_^+^ ions in the trap. CF_3_^+^ reacts with small alkanes by hydride abstraction. They are then detected as [M − H]^+^ [12,28].

O_2_^+^ is also used for the general detection of many compounds, alkanes among them. It is formed from dioxygen by electron ionization. It mainly reacts by charge transfer. O_2_^+^ is a non-specialized precursor as many molecules may react with it. However, it may induce important fragmentation.

Negative precursor ions may be formed as well. Very recently, we successfully used O^−^ and HO^−^ for halogenated compounds detection. The ICR trap can only contain either positive or negative charges. When using negative mode, the trapping potentials are set to a negative value. All negative species, including electrons, are then trapped. Electrons are ejected from the cell by applying a specific RF potential to the trapping plates. A packet of pure O^−^ is obtained by dissociative attachment of a low energy electron to N_2_O (circa 2–3 eV). O^−^ reactivity on halogenated compounds is complex. It may react by nucleophilic substitution, proton transfer, H_2_^+^-formal abstraction, or addition for hydrofluorinated olefins (HFO). Interestingly, for HFC134a (CF_3_CH_2_F) or HFC143a (CF_3_CH_3_), the only products are C_2_F_4_^−^ and C_2_HF_3_^−^, respectively, corresponding to H_2_^+^ formal abstraction leading to H_2_O. O^−^ precursor is then used for HFC detection.

Hydroxide anion is formed by a further step of the reaction of ammonia on an oxygen anion. HO^−^ mainly proceeds by a proton abstraction reaction, and reacts with any acidic compound whose gas phase acidity is lower than 1633 kJ mol^−1^ [29]. This precursor is quite complementary to the H_3_O^+^ precursor. For example, trihalomethanes (CHCl_3_, CHBr_3_, CHBrCl_2_, and CHBr_2_Cl) are ionized by HO^−^, but not by H_3_O^+^.

In some cases, it can be useful to use other proton transfer precursors than H_3_O^+^. On the low proton affinity side, H_3_^+^ can be produced by electron ionization and further reaction on H_2_. It will react with many more species than H_3_O^+^ (including N_2_ and H_2_O). On the high proton affinity side, many protonated molecules are available and can be produced by reacting H_3_O^+^ with the neutral. One example is protonated difluorobenzene; we have shown that it reacts quite efficiently with many VOCs and leads to less fragmentation than H_3_O^+^.

**Figure 5:**
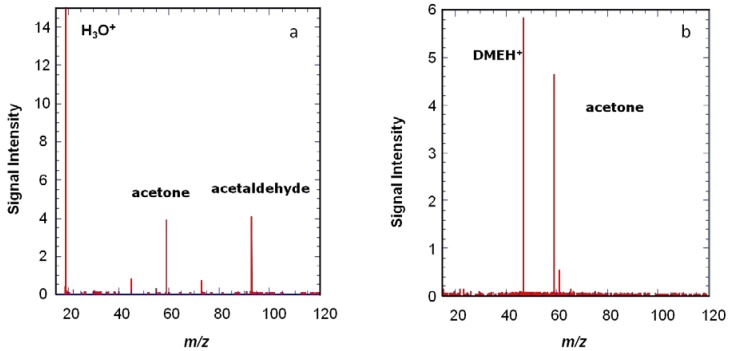
Reaction of two different ionic precursors with the same gas mixture, containing four VOCs: acetaldehyde, acetone, butanal, and toluene. (**a**) With the H_3_O^+^ ions all four VOCs are protonated. (**b**) With protonated dimethylether as precursor ions (produced by reacting H_3_O^+^ ions on a pulse of dimethyl ether), only protonated acetone at *m*/*z* 59 is observed, showing a much more specific ionization [30].

Another example is shown in Figure 5, where protonated dimethyl ether is shown to give a more specific ionization than H_3_O^+^. The use of several precursors with different proton affinities can be useful to help see species present at low concentrations.

When analyzing complex mixtures, it is interesting to switch between different precursors to identify as many products as possible. Switching between precursors is regulated by the software so as to alternate the different sequences and is illustrated below in Section 4.2.

## 3. Quantification

Chemical ionization is carried out in the ICR trap under well-controlled conditions. Direct quantification of the analytes is possible from peak intensities if pressure in the cell and reaction rate coefficients are known [27,31].

The intensities of the ions are given by the mass spectrum. They are measured relative to precursor ion intensity before the reaction. When several species are present, care must be taken that secondary reactions remain negligible. We always make sure that the intensity of the precursor ion signal has not decreased by more than 25% after reaction with the sample to be analyzed.

**Figure 6:**
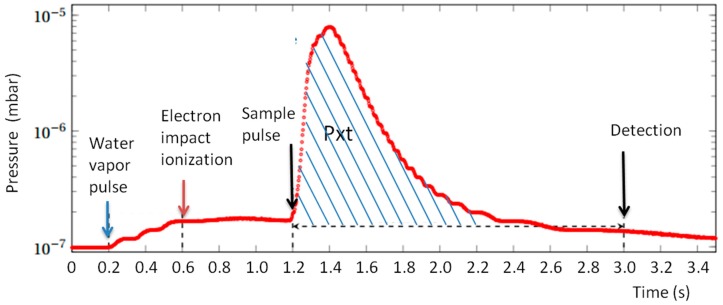
Temporal variation of the pressure during the sequence. The first pressure increase corresponds to the water vapor pulse, decreasing very slowly as the water sticks to the vacuum chamber walls. Then, the sample pulse gives a sharp pressure increase and at the time of detection, it has been pumped out. The Pxt value is obtained by integration of the pressure between the beginning of the sample pulse and the detection, minus the background (i.e., minus the integrated value when the sample pulse is not present). It corresponds to the hatched area on the graph.

Pressure in the ICR vacuum chamber is measured continuously using a Bayard Alpert gauge (Micro-ion plus, MKS Instruments, Munchen, Germany). The analog output of the gauge is digitized with a time resolution of 5 ms. A typical pressure variation in a sequence using two gas pulses (H_2_O and sample) is shown in Figure 6. From the recorded pressure data, a value called Pxt is evaluated by time integration of the pressure on the interval between the introduction of the gas sample and the detection. The background measured when the sample pulse is not present is subtracted to obtain a Pxt value proportional to the quantity of sample gas introduced.

Only exothermic reactions occur in the ICR cell, and relative ion intensities are controlled by kinetic parameters. For a fast ion-molecule reaction, the reaction rate coefficient is often in the 10^−9^ molecule^−1^ cm³ s^−1^ range. The reaction rate coefficient k_M_i__ of a specific reaction between a precursor and an analyte can be measured experimentally by fitting the precursor ion decay when introducing the pure analyte product. Moreover, for a rapid reaction such as proton transfer, it can often be considered that the reaction rate coefficient is equal to the collision rate coefficient k_C_: the reaction will take place at every collision between the precursor ion and a VOC (while all collisions with the main components of air will be non-reactive). The collision rate coefficient is evaluated from the dipole moment, polarizability, and mass of the precursors and molecule [11].

Quantification of the analytes is derived as follows when the precursor ions, for instance, H_3_O^+^, react with several VOC molecules M_i_:

H_3_O^+^ + M_i_ → M_i_H^+^ + H_2_O (9)

This reaction proceeds with a rate coefficient k_M_i__ and is the first order for the hydronium ion and VOC molecule M_i_. The hydronium ion can react with all M_i_ molecules and its decay is then:

(10) $$\frac{d\;\left[H_{3}O^{+}\right]}{\mathrm{dt}}=-\sum_{i=1}^{n}\;[k_{M_{i}}]\left[H_{3}O^{+}\right]=-S\left[H_{3}O^{+}\right]$$

With n being the number of molecules analyzed, [H_3_O^+^] and [M_i_] the concentrations in the molecule.cm^−3^, and:

(11) $$S=\sum_{i=1}^{n}k_{M_{i}}\;\left[M_{i}\right]$$

[M_i_] represents a concentration such as:

(12) $$\left[M_{i}\right]=\frac{N}{V}=\frac{P_{M_{i}}}{k_{b}T}$$

where P_M_i__ is the partial pressure of M_i_ in the gas stream (in Pa), T the temperature (in K), and k_b_ the Boltzmann constant.

While precursor ions decrease exponentially as a function of reaction time, the molecular ion of the analytes increases according to:

(13) $$\frac{d\;\left[M_{i}H^{+}\right]}{\mathrm{dt}}=k_{M_{i}}\left[M_{i}\right]\left[H_{3}O^{+}\right]$$

Integration of (10) over reaction time t_R_ gives:

(14) $$\left[H_{3}O^{+}\right]={\left[H_{3}O^{+}\right]}_{0}{\;e}^{-{\mathrm{St}}_{R}}$$

Using expression (14) in (13) and integrating between 0 and t_R_ gives:

(15) $$\left[M_{i}H^{+}\right]={\left[H_{3}O^{+}\right]}_{0}\frac{k_{M_{i}}t_{R}\;\left[M_{i}\right]}{S}\left(1-e^{{\mathrm{St}}_{R}}\right)$$

Elimination of S between (12) and (13) gives:

(16) $$\left[M_{i}H^{+}\right]=\left(1-\frac{\left[H_{3}O^{+}\right]}{{\left[H_{3}O^{+}\right]}_{0}}\right)\;{\left[H_{3}O^{+}\right]}_{0}\;\frac{k_{M_{i}}t_{R}\;\left[M_{i}\right]}{\ln\left(\frac{\;\left[H_{3}O^{+}\right]}{\;{\left[H_{3}O^{+}\right]}_{0}}\right)}$$

The total number of ions is a constant, but a small decrease can occur during the gas pulse. The sum of all ions is evaluated at the time of detection and we consider (H_3_O^+^) as the relative signal of H_3_O^+^ to total ion count, and (M_i_H^+^) as the relative signal of the protonated analyte relative to total ion count. In this derivation, secondary reactions are considered as negligible. To ensure that this is the case, we take care that the decrease of the precursor ion intensity after reaction with the sample gas pulse is less than 25%. From (14), we can express [M_i_] as:

(17) $$\left[M_{i}\right]=-\ln\left(H_{3}O^{+}\right)\frac{\left(M_{i}H^{+}\right)}{k_{M_{i}}\left(1-\left(H_{3}O^{+}\right)\right)t_{R}}$$

X_M_i__, the mixing ratio of M_i_ (number of M_i_ molecules divided by the total number of molecules), here expressed in ppm, is then given by:

(18) $$X_{M_{i}}\left(\mathrm{ppm}\right)=10^{6}k_{b}T\frac{-\ln\left(\left(H_{3}O^{+}\right)\right)\left(M_{i}H^{+}\right)}{k_{M_{i}}{P\;t}_{R}\;\left(1-\left(H_{3}O^{+}\right)\right)}$$

The value P.t_R_ is equal to the integrated value of the pressure observed during the experiment between gas sample introduction and detection, i.e., it is the Pxt value described previously.

**Figure 7:**
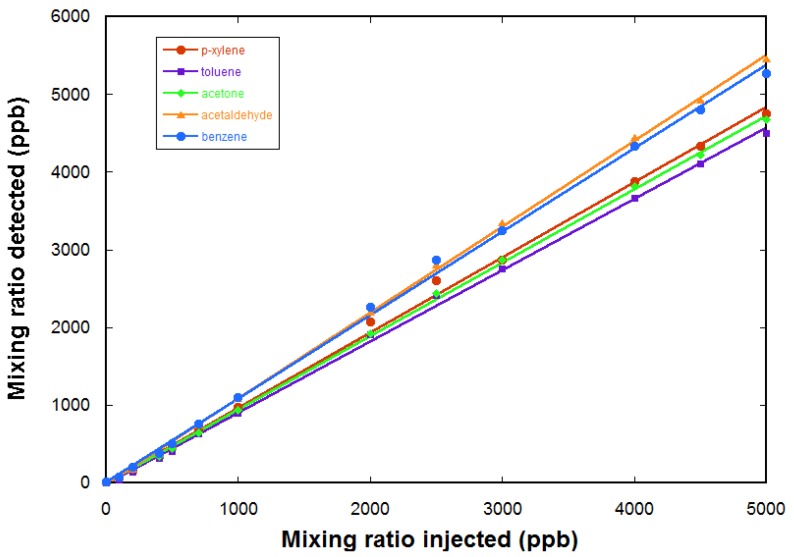
Determination of the mixing ratios with the FTICR-MS with H_3_O^+^ as the precursor ion using a calibrated gas mixture of five COVs diluted in air. For each point, the transient is accumulated over 25 sequences before doing the Fourier transform. The measured mixing ratios are determined using the ion intensities, the time resolved pressure measurement in the ICR cell, and experimental determinations of the rate coefficient for the chemical ionization reactions. The response of the FTICR-MS is linear.

Quantification is tested using calibrated gas mixtures. Here, we use a gas cylinder containing a calibrated mixture of five VOCs in air, each at a mixing ratio of 5 ppm. The five VOCs are acetaldehyde, acetone, benzene, toluene, and p-xylene. A further dilution in air zero is realized using a diluter (gasmix from Alytech, Juvisy-sur-Orge, France, fitted with two mass flow controllers). Figure 7 gives the measured mixing ratios for the VOCs as a function of the injected mixing ratio (determined by the dilution ratio in air zero). The response of the FTICR-MS instrument is linear. The limit of detection for these compounds is determined to be ~200 ppb for these compounds (from the noise level in a one sequence experiment).

## 4. Application to Real Time Measurements

Compact FTICR-MS associated with chemical ionization can be applied to real-time and on-site measurements in many fields, either for environmental applications (air pollution monitoring) or for industrial process control. Among the applications already carried out, it is possible to mention:

- analysis of exhaust gases from automotive engines (on engine test benches)
- monitoring of air purification by the air generator used in submarines
- study of air treatment using cold plasmas to remove VOCs
- real time analysis on VOCs emitted by thermal degradation of materials (polymers, food, …)

The first real time measurement example given below belongs to this last field. Additionally, the second one illustrates the successive use of different precursors.

### 4.1. Polymer Thermal Degradation

Thermal degradation of poly(methyl methacrylate) PMMA [C_5_H_8_O_2_]_n_ is a widely used transparent material that is recyclable [32,33]. Its thermal degradation is performed under a constant nitrogen flow (100 K min^−1^) with controlled temperature conditions [34]. The sample (3.7 mg) is heated in an oven with a constant temperature gradient (9.02 mg min^−1^) and the ion intensities of the VOC mixture emitted in the gas flow are followed in real time. Each VOC quantification described in Section 3 allows us to convert these intensities into mixing ratios, which can themselves be converted into massic emission rates since the gas flow and the molecular weight of each compound is known. The time scale is then converted to a temperature scale using the temperature gradient value.

**Figure 8:**
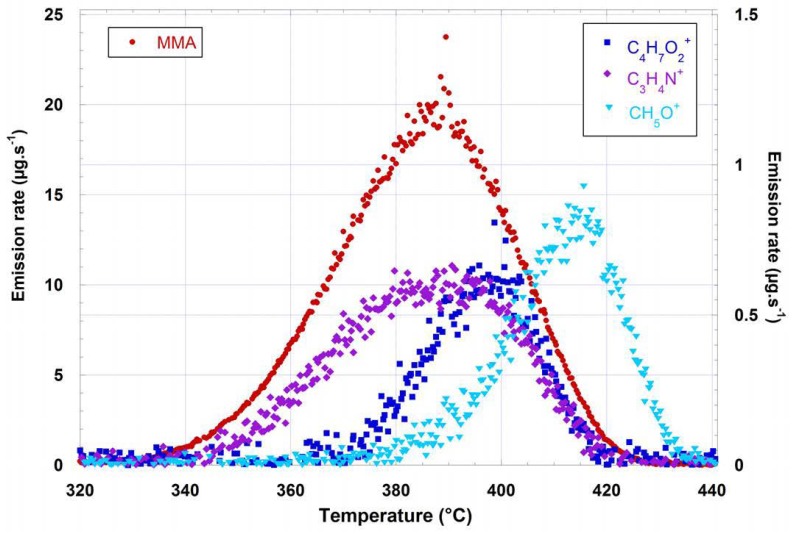
Variation of the emission rate of each VOC as a function of oven temperature, for a 3.7 mg PMMA sample heated under a nitrogen flow of 100 mL min^−1^. Left scale = MMA, right scale = emitted VOCs.

Figure 8 shows the resulting curves, giving the variation of the emission rate of each VOC with oven temperature.

The major VOC detected is MMA, as expected from well-known experimental and mechanistic studies [33]. The mass of MMA integrated on the emission peak was quantified to 79% of sample mass. Interestingly, other VOCs were detected as minor compounds: methanol CH_4_O (2.05%), ethyl methacrylate C_4_H_6_O_2_ (1.58%), and propene nitrile C_3_H_3_N (2.45%). The two former compounds can be explained by mechanistic considerations on PMMA degradation. The presence of propene nitrile is more surprising since PMMA does not contain nitrogen. Propene nitrile probably arises from a polymerization initiator, or from an additive in the polymer, probably after fragmentation.

### 4.2. Monitoring with Alternance of Several Ionic Precursors

**Figure 9:**
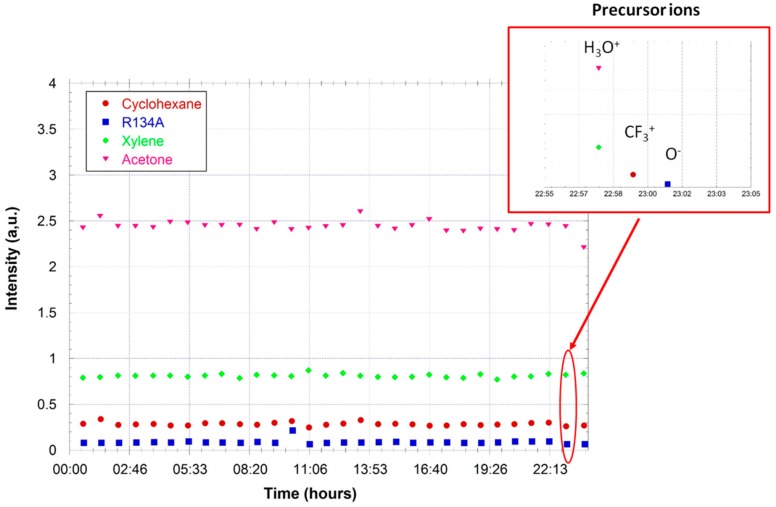
Monitoring of the gas injected in an air regeneration unit for a period of one day. Acetone and xylene are monitored using H_3_O^+^ as the precursor ion. The cyclohexane is monitored using CF_3_^+^ and O^−^ is used for the detection of Freon R134a. The zoom shows the temporal separation of the sequences using the three different precursors.

In the following example, the monitoring using three different ionic precursors (H_3_O^+^, CF_3_^+^, and O^−^) of a gas sample injected in an air regeneration unit is shown. The sample contains several products, simultaneously monitored with these precursors. Figure 9 shows the time monitoring of four of these compounds: acetone C_3_H_6_O, xylene C_8_H_10_, cyclohexane C_6_H_12_, and R134a Freon (1,1,1,2 tetrafluoroethane) C_2_H_2_F_4_. Each of them leads to a unique product ion for at least one precursor.

Acetone and xylene are both monitored with H_3_O^+^, which reacts with them by proton transfer.

H_3_O^+^ + CH_3_COCH_3_ → C_3_H_7_O^+^ + H_2_O (19)

H_3_O^+^ + C_6_H_4_(CH_3_)_2_ → C_8_H_11_^+^ + H_2_O (20)

Cyclohexane C_6_H_12_ is not ionized by H_3_O^+^, but evidenced using the reaction of CF_3_^+^ ions.

CF_3_^+^ + C_6_H_12_ → C_6_H_11_^+^ + CF_3_H (21)

R134a freon is analyzed using the O^−^ precursor.

O^−^ + C_2_H_2_F_4_ → C_2_F_4_^−^ + H_2_O (22)

This channel is dominant with a branching ratio of 99%, with the other ion produced being H_2_OF^−^ [35].

## 5. Conclusions and Perspectives

FTICR mass spectrometers based on permanent magnets are robust instruments that can be applied to field measurements of VOCs. Chemical ionization is implemented directly in the ICR cell.

Gases are introduced in a pulsed way, with a first gas pulse being used for the formation of the precursor ions and a second one for their reaction with the sample. H_3_O^+^ is the more widely used precursor ion as it reacts with most VOCs and leads to a detection limit of ca 200 ppb for one sequence. Other precursors can be used as it is easy to pulse gases from different introduction lines from one sequence to another. The sensitivity is improved compared to previous permanent magnet FTICR that had a limit of 1 ppm for one sequence, mostly due to the higher magnetic field. It can be further improved by accumulating transients over several sequences. The PTRMS instrument in which the chemical ionization is done at pressures close to 1 mbar reaches a lower detection limit, down to the ppt range [36]. However, the FTICR instrument also gives access to high concentrations, either by electron ionization or by adjustment of the sample gas pulse length or pressure when using chemical ionization.

The resolving power of the mass measurement allows a precise determination of the compounds present. The time evolution of their concentration can be followed with a time resolution of a few seconds.

The measurements presented in this paper are for VOCs present at trace concentration in air. However, the technique is applicable over a very large concentration range (up to concentrations in the % range) and can also be used for the detection of organic compounds in water using a semi-permeable membrane [37].

## 6. Patents

Two patents have been issued on the use of the magnetic structures described in 2.2 for mass spectrometry:

Permanent Magnet Ion Trap and Mass Spectrometer Using Such a Magnet (FR2835964, priority date 14 February 2002, inventors Bellec, G.; Boissel, P.; Heninger, M.; Lemaire, J.; Mauclaire, G.)

Ion Trap with Longitudinal Permanent Magnet and Mass Spectrometer Using Same (FR2874125, priority date 5 August 2004, inventors Heninger, M.; Lemaire, J.; Mauclaire, G.; Boissel, P.)
